# The role of sedimentation and natural compaction in a prograding delta: insights from the mega Mekong delta, Vietnam

**DOI:** 10.1038/s41598-018-29734-7

**Published:** 2018-07-30

**Authors:** Claudia Zoccarato, Philip S. J. Minderhoud, Pietro Teatini

**Affiliations:** 1 Department of Civil, Environmental and Architectural Engineering, University of Padova, Via Marzolo 9, 35131 Padova, Italy; 2 Utrecht University, Physical Geography, Utrecht, 3508 TC The Netherlands; 3 Unit of Soil and Groundwater Systems, Deltares, Delta Research Institute, P.O. Box 85467, 3508 AL Utrecht, The Netherlands; 4 Institute of Marine Sciences, National Research Council, Arsenale Tesa 104, Castello 2737/F, Venice, 30122 Italy

## Abstract

The Vietnamese Mekong Delta was formed by rapid transgression during the second half of the Holocene by deposition of mainly unconsolidated, fine-grained (clayey) sediments undergoing high compaction rates. The natural subsidence can seriously impact the already vulnerable delta plain as its low elevation exposes the delta to global sea level rise, flooding, salinization. Human activities such as groundwater pumping, infrastructural loading, sand mining and dam construction have exacerbated the effects of natural consolidation. Here we present a novel modeling study that has allowed to reproduce the formation and evolution of the Mekong delta over the past 4000 years. Using an adaptive finite-element mesh, the model properly simulates accretion and natural consolidation characterizing the delta evolution. Large soil grain motion and the delayed dissipation of pore-water overpressure are accounted for. We find that natural compaction of Holocene deposits following delta evolution exceeds predicted values of absolute sea level rise. The unprecedented high rates (up to ~20 mm/yr) threaten the lower delta plain with permanent inundation and inevitably reduce the designed service life of flood defense structures along the coast. Total subsidence and sediment delivery to the delta plain will determine its future elevation and vulnerability to relative sea level rise.

## Introduction

The populous Vietnamese Mekong Delta (VMD) hosts a thriving agricultural and industrial economy^1^. Like many deltas in the world^2^, its current delta plain has only been formed recently by deposition of sediments during the last several thousand years^3^. Its low elevation makes the VMD vulnerable to global sea level rise^4,5^, river flooding^6^, and salinization^7^. Moreover, fluvial sediment supply of the Mekong river is diminishing due to upstream dams^8–10^ and decreased activity of tropical cyclones over the Mekong river basin^11^. Within the delta, sediment delivery to the flood plains is decreasing due to dykes^12^ and sand mining^13,14^.

In addition, the delta is subsiding^15^ and the rates are accelerating due to the strong increase in groundwater extraction during the past decades^16^. Although human activities, like groundwater extraction and infrastructural loading, can increase land subsidence, sediment compaction in deltas is a natural process inherently related to delta evolution. As deltas evolve and prograde, new sediments are deposited on top of older, earlier deposits. The gravitational load of overlying sediments (overburden) causes the underlying sediments to compact (reduction in sediment volume and increase in bulk density), as a result of pore collapse (mechanical grain reorganization) and fluid expulsion^17^. Delayed dissipation of excess pore water pressure can result in ongoing compaction long after sedimentation has ceased. This process is especially apparent in fine-grained soils (i.e., peat and clay). The factors that determine the rate of natural compaction, and possibly land subsidence, are sediment type (hydrological and geotechnical properties) and the specific depositional history that has resulted in the present stratigraphy. Secondarily, compaction can be influenced by chemical or biological processes like dissolution, cementation, and decay of organic matter. As such, natural compaction can spatially be variable and characterized by high rates; in the Mississippi delta for example, compaction of Holocene sediments is identified as the main cause for delta subsidence, with multi-decadal rates exceeding 10 mm per year^18–20^. Determining compaction rates in modern transitional environments is difficult and direct observations and monitoring efforts are expensive and time consuming. Calculation of natural compaction is often complicated by the lack of data on sediment properties and incomplete knowledge of the depositional history^21^.

In VMD, very high compaction rates of Holocene strata between 25 and 41 mm per year are measured at three locations in coastal mangrove areas by surface elevation tables (SET) combined with marker horizons that register accretion at the surface^22^. Although sediment accretion for these locations exceeds compaction rate, which results in a net elevation gain of the surface, these numbers reveal the potential of the Holocene deltaic sediments to contribute to VMD subsidence through natural consolidation. This holds especially in cases where sediment accretion is reduced by natural causes, dyke development or following cultivation^23^. With subsidence posing an increasing threat to the VMD, it is essential to quantify the contribution of each specific driver to the delta subsidence to create an action perspective for sustainable delta management^16^.

In this paper, we aim to address and quantify natural compaction of the Holocene strata in the VMD as a result of the sedimentation history following delta evolution. We present a novel modeling approach that allows us to reproduce the VMD evolution and quantify the corresponding compaction over the past 4000 years. The model represents the most low-lying and vulnerable part of the delta, mainly consisting of fine-grained Holocene material (clays). Due to the extremely high porosity of newly deposited soil, the medium is deformable with large solid grain movements. Therefore, we use a two-dimensional (2D) groundwater flow model coupled to a one-dimensional (1D) compaction module where the assumption of infinitesimal displacements is relaxed to account for large deformations^24^. This implies the recast of Darcy’s law in term of relative velocity of the soil grains to the fluid velocity. We do not incorporate chemical or biological processes as they represent secondary factors contributing to compaction because of general waterlogged conditions^25,26^. This approach may serve as a model to investigate natural compaction in other deltas and prograding coastal environments elsewhere in the world.

## Results

**Figure 1:**
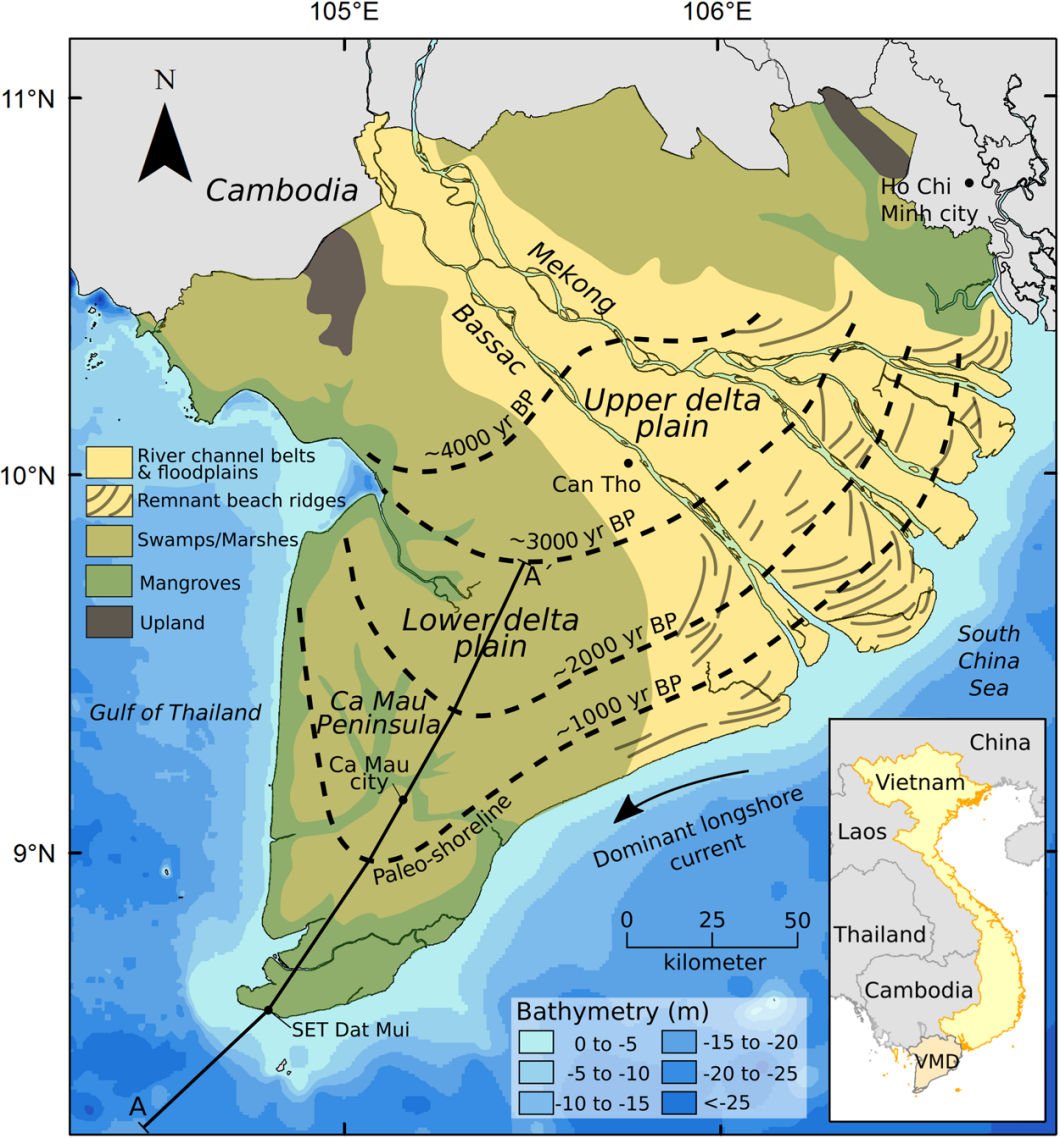
Map of the Vietnamese Mekong Delta (VMD) in Southeast Asia showing the main depositional paleoenvironments^52^. Dashed lines represent the approximated paleo-coastlines based on a combination of data^3,41,42^. The 3000 yr BP shoreline demarcates the boundary between the upper and lower delta plain. The A-A′ profile is the selected representative transect along which the 2D model is applied for the simulation of the delta progradation. Ocean bathymetry ‘World Ocean Base’ map from NOAA and Esri.

**Figure 2:**
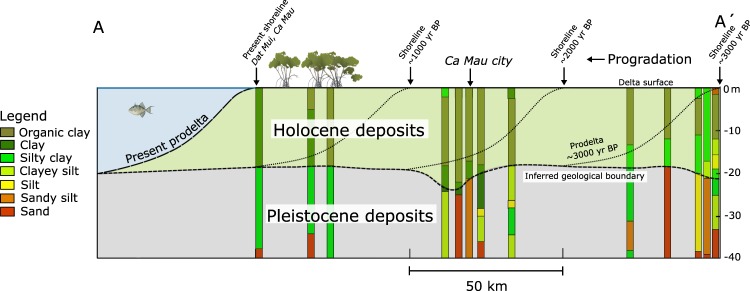
Lithological borelogs of the Division of Water Resources Planning and Investigation for the South (DWRPIS) of Vietnam and Division for Geological Mapping for the South (DGMS) of Vietnam along the transect A-A′. The boundary between the Holocene deposits, mainly consisting of organic clays (clays with high organic matter content), clays and silty clays and the underlying coarser and stiffer Pleistocene deposits is highlighted. The prodelta gradient is determined by profile measurements of the submarine delta front at the west side of the present VMD. The north part of the section A-A′ demarcates the present boundary between the lower and the upper delta plain^28^. The lithological profile at Dat Mui has been obtained from Surface Elevation Table (SET)^29^.

The present study focuses on the simulation of the VMD evolution over the past 4000 yrs during which the prodelta moved 200 km in seaward direction along the alignment A-A′ traced in Fig. 1. Large amounts of fine-grained material from the Mekong river mouths entered the sea and were subsequently transported by dominant longshore currents in southwest direction. Accumulation of these sediments resulted in a shoreline migration of about 150 km seaward during the past 3000 yrs at an average progradation rate of 50 m/yr^27^, creating the Ca Mau peninsula. The Holocene sediments mainly consist of clay and organic clay and accumulated to a total thickness varying between 18 and 25 m on top of the older Pleistocene deposits (Fig. 2). We assess spatio-temporal compaction and deformation of the Holocene sediments following delta progradation for both the prodelta and the lower delta plain. The northern part of section A-A′ demarcates the boundary between the marine dominated deposits of the lower delta plain and the fluvial dominated deposits of the upper delta plain^28^ (Fig. 1).

### Prodelta formation and progradation

The large accumulation of clayey sediments on the subaqueous shoreface (i.e. prodelta) caused the rapid progradation of the delta in the direction of profile A-A′. The prodelta migration of 50 m/yr is simulated by a spatio-temporal evolution of the sedimentation rate properly constrained by available data (see Materials and Methods).

The modelling approach enabled the dynamical simulation of the prograding prodelta employing an adaptive mesh. New grid elements were progressively added at increasing time steps to account for the accumulation of new material, burying the underlying sediments. The mesh initially consisted of 1000 nodes and 998 triangular elements, which increased during the simulation to the final values of 103,799 nodes and 206,372 triangles. The initial thickness of a new-deposited element equaled 0.2 m, whereas a 400 m-discretization was used along the x-axis with a 1:1000x-scaling factor. The mesh elements deformed accordingly to the occurring consolidation process and their thickness decreased as the load of the overlying sediments increased. The deformations were obtained through the computation of the movement of the soil grains, which are represented by the grid nodes in the 1D geomechanical model^24^.

**Figure 3:**
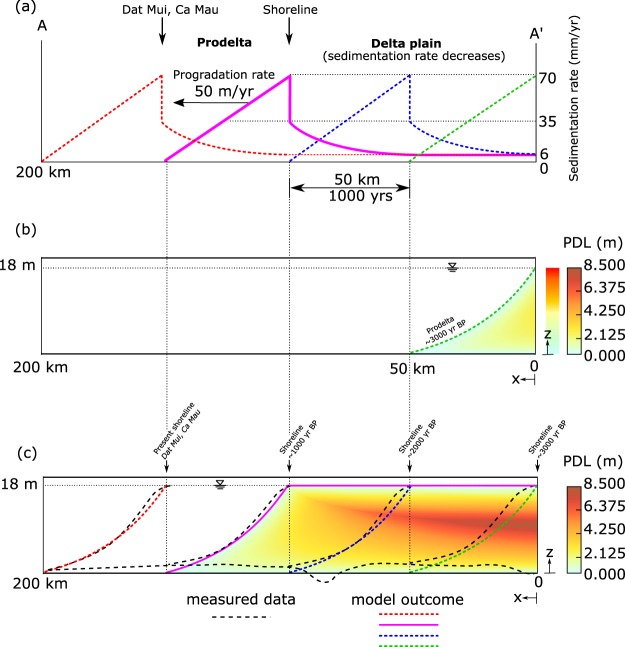
Prodelta formation, progradation and evolution of the delta plain. (**a**) Profiles of the sedimentation rate at 3000, 2000, 1000 yrs BP and present. The information on the sedimentation rates are available from literature data^22,28,42,43^. (**b**) and (**c**) PDL distribution after the prodelta formation at 3000 yrs BP and during the delta plain evolution at 1000 yrs BP, respectively, resulting from sediment accretion and compaction. The PDL value at any *z*-coordinate represents the compaction of the sediment column underlying *z* from time of deposition to the actual time. In subpanel (**c**), the prodelta profiles at 3000, 2000, 1000 yrs BP and present from the model outcome are depicted by colored lines whereas dashed-black lines represent the reconstructed profiles (Fig. 2). The prodelta evolution is the consequence of the sedimentation rates given in subpanel (a).

Prodelta formation was simulated with a sedimentation rate (*ω*) ranging from 0 to 70 mm/yr with shoreline proximity, accounting for increasing near-coastal sedimentation. This sedimentation evolved over the distance of 50 km in 1000 yrs (Fig. 3a). After 1000 yrs, i.e. at 3000 yrs BP, the prodelta was completely formed. At this stage, the accumulated sediments became elevated above sea level and the depositional environment changed from prodelta to lower delta plain. The simulated thickness of compacting sediments at the coastline reached 18 m (Fig. 3b). This value is in line with the 18–20 m-thick Holocene clays on top of stiffer Pleistocene silty clays to silty sands reported for the Ca Mau peninsula (Fig. 2)^28,29^. A detailed description of the data used to derive the *ω* behavior is provided in Materials and Methods section.

Figure 3b shows the post-depositional lowering (PDL) of the delta sediments occurred in the interval between 4000 and 3000 yrs BP. The PDL value at any *z*-coordinate represents the compaction of the sediment column underlying *z* from time of deposition to 3000 yrs BP and its integral over *z* gives the total compaction of the soil column. The highest PDL equal to ~2.3 m is experienced by sediments at the shoreline and approximately in the middle of the column thickness. As the cumulative amount of unconsolidated sediments deposited at the shoreline is 35 m (average sedimentation rate over 1000 yrs is 35 mm/yr), the total compaction taking place during prodelta formation is 17 m, corresponding to an average rate of 17 mm/yr.

After 3000 yrs BP, the prodelta successively advanced along the delta progradation-direction at a constant speed of 50 m/yr until it reached the present 200 km-length. Figure 3c shows the profiles of delta progradation at 3000, 2000, and 1000 yrs BP, and at present. The model outcomes agree with: (i) data from corings that reveal the southward evolution of the delta and approximate locations of the prodelta in the past (Fig. 2); and (ii) high-resolution, offshore seismic profiles of the low-gradient prodelta surrounding the modern VMD with a maximum thickness of ~20 m at the shoreline^30,31^.

### Lower and upper delta plain evolution

**Figure 4:**
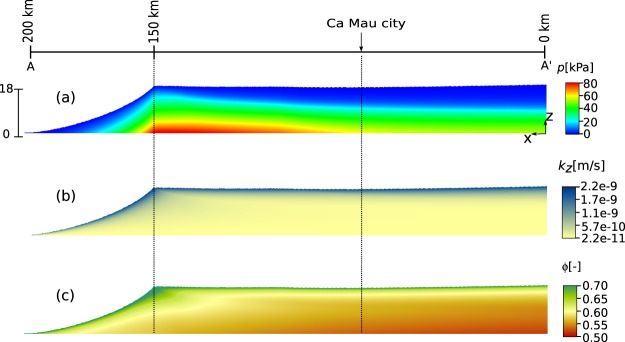
Distribution of (**a**) overpressure, (**b**) permeability, and (**c**) porosity within the cross-section A-A′ (see Fig. 1) at present after calibration with available data of sedimentation rates and hydro-geomechanical properties of the Holocene deposits.

During delta evolution over the past 3000 yrs, the lower delta plain remained about constantly elevated at the sea level or slightly above as it is at present, with wetlands forests and marshes being the main eco-morphological features^3^. This is the result of a dynamic balance of sediment accumulation and compaction. Accommodation created by ongoing compaction following delayed overpressure dissipation was filled by new, clastic, and organic sediments. As a result, the delta plain sustained its elevation and the total thickness of Holocene sediments remained constant, while becoming more compact. Simulated sedimentation rates are based on available data (see Materials and Methods). The maximum sedimentation rate equals 35 mm/yr just behind the shoreline, corresponding to the location of Dat Mui at the present day coastline (Ca Mau peninsula, see Fig. 2). The sedimentation rate decreases progressively moving inland along the cross-section A-A′ (Fig. 3a) as, with the gradual decrease in overpressure (Fig. 4a), compaction rate decreases and less sediment is needed to fill the accommodation. On the upper delta plain, a constant sedimentation rate of 6 mm/yr suffices to counterbalance the consolidation (Fig. 3a).

At 1000 yrs BP (Fig. 3a, magenta line), the total compaction is equal to 17 m at shoreline location (*x* = 100 km) and 37 m at the edge of the lower delta plain (*x* = 0 km), where the total thickness of the deposited material (before consolidation) is 35 m and 55 m, respectively (Fig. 3c). The maximum PDL at 1000 yrs BP amounts up to 8.5 m (*x* = 0 km, location of the shoreline 3000 yrs BP) (Fig. 3c), meaning that these sediments are now buried 8.5 m below their elevation at time of deposition. This value increases to 9.3 m at present, i.e., at the end of the model simulation.

The overpressure (*p*) in the present condition (*t* = 0 yrs BP) is shown in Fig. 4(a). The maximum value (*p* = 80 kPa) is located at the bottom of the Holocene column at the shoreline (*x* = 150 km) where sedimentation rate assumes the largest value. Overpressure is lower seaward as there is less overburden and inward as partial dissipation of overpressure has already taken place. At the edge of the lower delta plain (*x* = 0 km) the remaining overpressure at the bottom of the sediment column amount to *p* = 53 kPa. Obviously, there is no overpressure at the delta surface. At Ca Mau city (Fig. 4), the simulated maximum overpressure is equal to ~60 kPa and the maximum PDL to ~7.8 m, suggesting that natural compaction is an ongoing process. The modeled overpressure values are within the range of values measured in shallow VMD clays using cone penetration tests (overpressures up to 200 kPa are reported)^32,33^. Figure 4(b) and (c) show the vertical permeability (*k*_*z*_) and porosity (*ϕ*) distribution within the domain, respectively. Porosity decreases with depth due to the increase of the intergranular effective stress (*σ*_*z*_), see Materials and Methods for the *ϕ*-*σ*_*z*_ relationship. An empirical relationship is used to relate permeability to soil deformation (see Materials and Methods) and, in turn, to porosity distribution. The permeability reduction of about two orders of magnitude from the shallowest to the deepest deposits strongly delays overpressure dissipation and, consequently, consolidation dynamics.

### Sensitivity analysis

The model is calibrated using the available datasets of sedimentation rates, geotechnical soil properties and lithology of the Holocene sediments (see Materials and Methods). Although the model results provide a satisfactorily match with observations, we present a sensitivity analysis of the model output to the main input data and parameters to evaluate the variability range of the system dynamics due to different factors. The sensitivity analysis refers to the prodelta formation phase, i.e., the time interval between 4000 and 3000 yrs BP.

**Figure 5:**
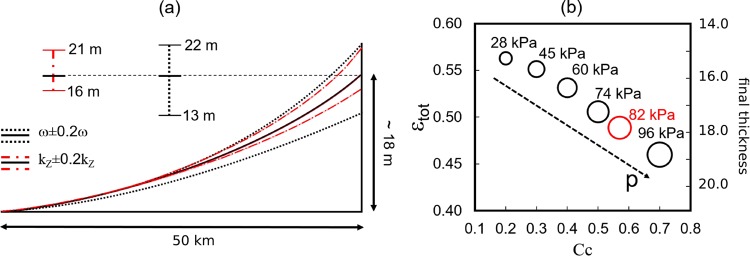
Influence of model parameters on the total elevation of the Holocene sediments at *t* = 3000 yrs BP. (**a**) A 20% variation of sedimentation rate, *ω*, and the vertical permeability, *k*_*z*_ is investigated. The black solid line represents the shoreline computed through the calibration values used in the model, whereas the black dot-dot and red dot-dashed lines show the variability of the model outcome. (**b**) Total deformation, *ε*_*tot*_, of the soil column at position *x* = 0 m for different values of the compression index, *C*_*c*_. The overpressure *p* increases at increasing *C*_*c*_. The red circle refers to the calibrated scenario.

The investigated range of model parameter (*m*_*p*_) variability is 20% (${m}_{p}\pm \mathrm{20 \% }{m}_{p}$), which is probably a somewhat narrow range due to the large uncertainty associated with the hydraulic permeability and deposition rate. Figure 5(a) shows the changes in total elevation of Holocene deposits due to variations of permeability and sedimentation rate. A smaller vertical permeability implies a slower overpressure dissipation in time, thus lower consolidation rate and higher elevations. A variation in the total elevation of 12–15% is found for *k*_*z*_ ± 20%*k*_*z*_. On the other hand, keeping permeability fixed, the sedimentation rate affects the thickness of deposited sediments, causing an approximate 22% difference in elevation from the calibrated value.

The relation between sediment deformation and the geomechanical properties of the soil is also investigated, with the results presented in Fig. 5(b). The geomechanical characteristics are described through the coefficient of compression, *C*_*c*_, with organic clays having higher compressibility than mineral clays. *C*_*c*_ is kept constant for each lithological unit. A smaller *C*_*c*_ yields lower deformations of the porous matrix, which, in turn, influences the permeability distribution within the soil column. Following the relationship between permeability and vertical deformation *ε*_*v*_ provided in Materials and Methods, a 50% decrease of vertical deformation results in a 23% higher permeability. As such overpressure dissipation is higher, subsequently larger consolidation rates are computed. This complex behavior is summarized in Fig. 5(b) where the total deformation of the soil column *ε*_*tot*_ at *x* = 0 km and *t* = 3000 yrs BP is plotted against *C*_*c*_. *ε*_*tot*_ is computed as *ε*_*tot*_ = (*T*_*tot*_ − *T*_3000_)/*T*_*tot*_ with *T*_3000_ the thickness of the Holocene column at *t* = 3000 yrs BP and *T*_*tot*_ the thickness of the deposited, unconsolidated material between *t* = 4000 yrs BP and *t* = 3000 yrs BP. Overpressure drops of about 70 kPa follow the reduction of *C*_*c*_ from 0.7 to 0.2. This implies a 20% variation of *ε*_*tot*_, corresponding to 3.6 m-difference in elevation.

### Future delta evolution

In the past, natural compaction of the delta plain was counterbalanced by clastic and organic sedimentation, allowing the delta to maintain its elevation above sea level. However, cultivation^23^, dyke development on the delta plain^12^, and reduction of upstream sediment supply have disturbed this natural balance. As a result, Ca Mau peninsula is receiving a decreasing amount of sediments both at the prodelta and on the delta plain. The feedback of this new condition on land subsidence is not straightforward because a dynamic coupling between accretion and overpressure dissipation, and therefore land subsidence, exists. Moreover, the coupling is governed by the intrinsic hydro-geomechanical properties of the accreting and underlying deposits.

**Figure 6:**
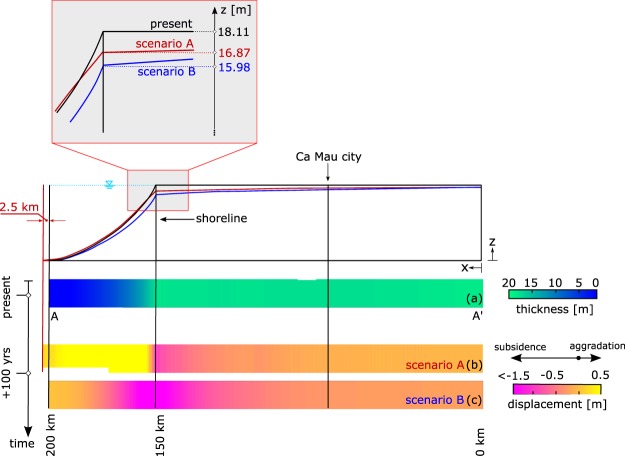
(**a**) Present thickness of the Holocene deposits along the transect A-A′ as obtained by the numerical model. (**b**,**c**) Land displacements along the A-A′ transect as computed by the model over the next 100 years for two sedimentation scenarios. In scenario A, sedimentation rates are halved (50%) compared to the calibrated distribution (Fig. 3b). In scenario B, sedimentation has completely stopped (*ω* = 0). Negative values mean land subsidence, positive aggradation.

To understand this complex mechanism the proposed model has been applied. We simulated two scenarios of delta evolution over the next 100 yrs. The first scenario (scenario A) investigates the situation in which the sediment supply is halved compared to the present values for which compaction and sedimentation are in equilibrium. The model results are provided in Fig. 6. With a 50% reduction of sedimentation rate, we can reasonably assume that the delta is still prograding at halved speed of 25 m/yr. The consequence is a net aggradation at the prodelta up to 6 mm/yr. However, on the delta plain, sedimentation is no longer enough to counterbalance the consolidation of the underlying deposited sediments. At the present shoreline location (Dat Mui) this results in an average subsidence rate of 12 mm/yr, amounting to ~1.2 m by the end of the century (Fig. 6b). Subsidence rates decrease gradually inland to 0.8 mm/yr towards the upper delta plain.

In the worst case (scenario B) sediment deposition is assumed to cease completely (*ω* = 0 mm/yr). Land subsidence is no longer counterbalanced by sedimentation and, because of the ongoing overpressure dissipation, subsidence is expected along the entire prodelta and delta plain. The results presented in Fig. 6c show a maximum loss of land elevation equal to ~2.0 m at the present shoreline, which means an average subsidence rate of 20 mm/yr. This rate gradually decreases toward the upper delta plain, where the subsidence rate drops to 2.4 mm/yr. Progradation of the VMD completely stops in this scenario and a large part of the lower delta plain will sink below sea level before the end of the century.

## Discussion

The main processes controlling delta progradation are sediment aggradation and natural compaction of Holocene sediments. Here natural compaction involves the volume reduction of the deposited material due to the loading exerted by the overlying, more recent deposits following the consequent overpressure dissipation. Our novel model allowed for the first time to simulate the spatio-temporal formation and evolution of the VMD along a representative 2D vertical section (Fig. 1) by using an adaptive mesh, with changing element shape and number following new sediment deposition and consolidation over time. The modelling application is properly supported by geomorphological and geotechnical information.

We estimate rates of natural compaction of the Holocene sediment sequence at the coastline equal to ~20 mm/yr as a result of thousands of years of VMD delta evolution. These high rates were previously unaccounted for^15^ and pose a serious threat to the Mekong delta as these rates cannot be mitigated. Moreover, such natural compaction rates directly affect unfounded structures and inevitably reduce the designed service life of coastal defense structures that are currently being build. As a result, if not taken into account, natural compaction seriously threatens Vietnam’s investments in a hard coastal protection system of the VMD coastline. Such measures should be considered carefully, also because the construction of a coastal dyke system decreases sedimentation on the hinterland, accelerating delta plain subsidence further.

Land subsidence in modern deltas is partly related to the decrease of sediment availability^2^. It is reported that the VMD has experienced a decrease of sediment supply by 12% in the 20th century. Larger reduction are expected in the coming decades. Dam development in the Mekong drainage basin may potentially cause major changes in the amount of sediment transported to the delta^34^. Major dam construction began in the Mekong basin in 1993; by April 2016, 35 dams had been commissioned for hydropower, irrigation reservoirs, and drinking water supply. A further 226 dams are under construction and even more are planned^35^. A recent study predicts that the cumulative suspended sediment in the Mekong river decreased to 51% following the construction of the current dams, and that this value goes up to 96% in case all planned dams are constructed^9^. Changing global climate may also affect the future water and sediment supply to the Mekong Delta^36^, as a large fraction of the suspended sediment load reaching the delta is associated with rainfall from tropical cyclones. Climate models suggest the locations of cyclone tracts to shift away from the latitude of the Mekong Basin, which may also lead to additional suspended sediment reduction^11^.

We estimate that a sediment reduction of 50% over the 21st century results in a loss of elevation amounting to ~1.2 m at the present coastline due to ongoing natural compaction of the Holocene sequence. The prediction worsens if sedimentation on the delta surface is completely prevented, leading to an elevation loss of ~2.0 m by the end of the century. More inland, towards the upper delta plain, the elevation loss reduces to ~10 cm and ~20 cm for a sediment reduction of 50% and 100%, respectively. Our estimates suggest that Ca Mau city will subside ~34 cm as a result of natural compaction of Holocene sediments during the next century as flood sedimentation in the city has completely ceases.

Other natural and human-induced drivers and processes may also contribute to subsidence and the future evolution of deltaic regions. Examples of natural subsidence drivers are tectonics, natural compaction of Quaternary units, and glacial isostatic adjustment. In the VMD their contribution to subsidence has been quantified in the order of a few mm/yr over the late-Holocene^3,37^. Therefore, they have played a secondary role on the recent delta evolution and have been neglected in our analysis. The effect of biological and chemical processes, which may trigger additional subsidence, have been negligible in the past as a result of the general waterlogged conditions and were therefore not included in this study. However, they should be accounted for in future modelling as drained conditions may be established in deltaic-plain sediments for agricultural purposes. Aeration triggers oxidation of the organic matter, which represents a large fraction of the top soils, and consequently enhances the ongoing natural compaction as experienced for example in the Rhine-Meuse (The Netherlands)^38,39^ and Po (Italy)^25^ river deltas. Concerning anthropogenic subsidence, groundwater pumping from the deep multi-aquifer system has significantly contributed, in the range of 10 to 25 mm/yr^16^, to the present land subsidence in the VMD over the last few decades. Oil and gas activities are also on the rise offshore of the VMD with areas undergoing seismic exploration and some initial drilling by the Vietnam National Oil and Gas Group (PetroVietnam) and international partners^34^. Future hydrocarbon explorations may contribute as well to subsidence of the delta.

Finally, also sea level rise contributes, although secondarily, to the relative loss of land elevation with respect to the sea level. Estimates of absolute sea level rise for the VMD are ~3.5 mm/yr^40^, which are small compared to the land subsidence the delta may experience in the coming decades. Our study reveals that natural compaction of the Holocene sediments alone can create subsidence rate up to one order of magnitude larger than absolute sea level rise.

As the VMD is only elevated ~1–2 m above sea level, the anticipated subsidence rates seriously threaten the lower delta plain with permanent inundation. Therefore, sediment supply to the delta plain and a proper management of surface water and groundwater resources are key factors for the VMD survival. The model presented here represents a valuable tool for understanding the natural compaction dynamics of the VMD and, more generally, of coastal environments at risk of submersion. It can be used to identify areas vulnerable to high compaction rates and hereby contribute to improve coastal protection plans.

## Materials and Methods

### Site evolution over the Holocene

With an area of 50,000 km^2^, the Mekong delta, largely situated in the southwest of Vietnam, is the third largest delta plain in the world and it is characterized by the largest areal extent elevated less than 2 m above mean sea level^2^, i.e. more than 20,000 km^2^ (Fig. 1). The combination of high sediment supply, wave-sheltered position and relatively shallow sea favored a very rapid growth of the delta over the last 6000 years^27^. Around ~3000 yrs BP, the delta changed from a tide dominated delta to a wave-tide dominated delta with increased long-shore sediment transport^28^. On entering the flat delta plain, the Mekong river branched out in eastward direction, forming a river landscape with large channel belts and floodplains. Sand transported by the river was deposited in sequences of wave-formed beach ridges between the different river mouths^41^. The impressive muddy load of the Mekong river was transported by wave-induced longshore currents towards the south-western part of the delta. The accumulation of fine-grained sediments filled the space between the coarser-grained Pleistocene surface and the present day delta surface. This resulted in the rapid progradation of the so-called Ca Mau peninsula (Fig. 1), and the formation of vast swamp, marsh and mangroves areas^14^.

### Available data to constrain the sedimentation and consolidation model

The prodelta moved seaward approximately 200 km during the past 4000 years^3,27^. This results in an average progradation rate of ~50 m/yr for the Ca Mau peninsula. Figure 1 shows a map of the present Vietnamese Mekong delta and its evolution in time. The model simulates the delta formation and progradation along the A-A′ section (Fig. 1) over the 4000 yrs-interval. Note that the 200 km-delta includes 50 km of prodelta.

The information on the sedimentation rates over the delta is available from the literature. In particular, the bay and prodelta sediments are found to accumulate at a rate up to 32–63.7 mm/yr^28,42^. Sedimentation rates up to 36.8 ± 3.1 mm/yr and 67.8 ± 6.6 mm/yr, respectively, at Dat Mui and the Bassac river mouth, have been quantified using marker horizon measurements at SET sites^22^. These data allow constraining the model input in term of prodelta sedimentation rates. An average sedimentation rate (*ω*) of 35 mm/yr over a 1000-yr time interval roughly corresponds to a prodelta progradation of 50 km.

For the lower delta plain we assume a dynamic balance between natural compaction and sedimentation, meaning that accommodation is filled through sedimentation but sedimentation rate does not exceed compaction rate. An average subsidence rate of 30–35 mm/yr is derived from 12 SET stations established at 4 locations in mangrove areas along the coast^22^. Thus, sedimentation rates of 30–40 mm/yr represent a likely estimate of the amount of sediment required at the coastal front to balance subsidence. Notice that these SET combined with marker horizon data are not representative of most of the the peninsula which is nowadays predominantly agricultural. Therefore, a reduced value is prescribed at the upper delta plain with an average floodplain sedimentation of ~6 mm/yr^43^. This results in a flat and lowly elevated surface in agreement with the present setting of the VMD lower delta plain. Sedimentation likely decreases gradually away from the coastline towards the upper delta plain. Following this principle and data to constrain on past coastline progradation, a consistent behavior of sedimentation rate, *ω*(*x*, *t*), for the prodelta and the delta plain was determined (Fig. 3a).

Surely, we are aware that the sediment deposition and its spatial variability as provided in Fig. 3a is a semplification of a much more complex process of sediment re-distribution, starting from the sources (the Mekong river and its mouth) to the dispersal work carried out by dominant longshore currents and river floodings in the delta plain. However, an accurate representation of these processes is beyond the scope of our study and it cannot be captured in a 2D modelling framework as the one used in the proposed analysis.

### Hydro-geomechanical data and model set-up

The main input parameters of the model are the hydro-geomechanical properties of the Holocene deposits. These features have been quantified using datasets from the geotechnical measurements summarized in Table 1. Lithological boreholes and geotechnical profiles in Ca Mau revealed the presence of very soft organic clays overlying soft mineral clays in the upper 20 m-depth^29,44^. Based on the available lithological information (Fig. 2), these two sediment types, which prevail within the Holocene sequence, are accounted for in the model.

**Table 1:** Measured ranges and values of the hydro-geomechanical parameters used in the simulations. The number of available measurements is indicated with *n*.

Parameter	Measured values	Modeled values
***Organic clay***		
Initial void ratio (*e*_0_)	2.52^44^	2.50
Compressibility index *C*_*c*_/(1 + *e*_0_)	0.21–0.43 (*n* = 10)^32^	0.30
***Clay/silty clay***		
Initial void ratio (*e*_0_)	1.55–2.63 (*n* = 133)^44^	1.89
Compressibility index *C*_*c*_	0.52–2.63 (*n* = 133)^44^	0.57
***Organic clay & Clay/silty clay***		
Initial permeability (*k*_*z*,0_)	2.2 ⋅ 10^−9^ m/s^32^	2.2 ⋅ 10^−9^ m/s
Anisotropy *k*_*x*_/*k*_*z*_	2.0^48^	2.0

The behavior of the vertical oedometric compressibility *c*_*b*_ versus the vertical effective intergranular stress *σ*_*z*_ represents the fundamental constitutive relationship implemented in our modelling approach. This law is obtained by integrating the relation:

1 $$\frac{-1}{\mathrm{(1}+e({\sigma }_{z}))}de={c}_{b}d{\sigma }_{z}$$

with *e* the void ratio and *e*(*σ*_*z*_) = *e*_0_ − *C*_*c*_log*σ*_*z*_. The void index *e*_0_ is representative of the porosity of the newly deposited sediments on the delta surface. An initial void index *e*_0_ = 2.5 has been obtained by geotechnical analyses on shallow organic clay samples collected in Ca Mau^44^. A corresponding *C*_*c*_ = 1.05 is computed using the relationship $\frac{{C}_{c}}{1+{e}_{0}}$ = 0.3 experimentally derived from the geotechnical surveys carried out by NGI^32^. These values are supported by empirical relationships^45,46^ and measurements on similar, shallow very-soft clays in Ho Chi Minh city where *C*_*c*_ = 1.01 with *e*_0_ = 2.23^47^. Different values of *e*_0_ and *C*_*c*_ are measured for the mineral clays underlying the organic clays. An average value *C*_*c*_ = 0.57 is derived using a liquid-limit relationship^45^ based on the analyses of 133 soil samples collected at Ca Mau city with *e*_0_ = 1.89. Notice that in the proposed model the geomechanical properties depend on the lithotype and vary with the vertical effective stress differently for each sediment type. Moreover, it has been assumed that the properties of each element shift from organic to mineral clay for an effective stress larger than 5 KPa, i.e., when the element is buried at depth approximately larger than 1 m.

Oedometer tests carried out by the NGI at zero-volume change provide a vertical hydraulic conductivity *k*_*z*,0_ = 2.2 ⋅ 10 ^−9^ m/s^32^. This value is considered representative for the unconsolidated soil at the delta surface. Moreover, the same tests also define the following relationship between *k*_*z*_ and the volumetric change *ε*_*vol*_ of the porous media due to compaction:

2 $$\frac{\mathrm{log}\,{k}_{z\mathrm{,0}}-\,\mathrm{log}\,{k}_{z}}{{\varepsilon }_{vol}}=4.0$$

Since *ε*_*vol*_ is provided by the numerical model as described below, Equation 2 allows reproducing the change of *k*_*z*_ during the delta evolution. Finally, an anisotropic hydraulic conductivity with a ratio between the horizontal value *k*_*x*_ and *k*_*z*_ equal to 2.0 is assumed for shallow clay material^48^.

### Governing equations

The formation and evolution of the VMD is described with the aid of a numerical model (NATSUB-2D) by coupling a 2D groundwater flow model over a cross-section of the delta and a 1D geomechanical module. The model allows describing the spatio-temporal evolution of the consolidation process within the forming delta system, depending on the overpressure evolution.

The rigorous equations of the 1D flow in an elastic saturated compacting porous medium was originally developed in the late 70 s^49,50^, where the hypothesis of infinitesimal displacement of the grains is relaxed and large soil deformations are accounted for introducing a geometric non-linearity. In a 2D vertical cross-section, the governing equations of the groundwater flow can be written as^24^

3 $$\frac{\partial }{\partial x}(\frac{{k}_{x}}{\gamma }\frac{\partial p}{\partial x})+\frac{\partial }{\partial z}(\frac{{k}_{z}}{\gamma }\frac{\partial p}{\partial z})=({c}_{b}+{\varphi }\beta )Dp-2\beta {k}_{z}\frac{\partial p}{\partial z}-\beta \frac{{k}_{x}}{\gamma }{(\frac{\partial p}{\partial x})}^{2}-\beta \frac{{k}_{z}}{\gamma }{(\frac{\partial p}{\partial z})}^{2}$$

where *k*_*x*_ and *k*_*z*_ are the horizontal and vertical hydraulic conductivities, *γ* is the specific weight of water, *c*_*b*_ is the soil oedometric compressibility, *ϕ* is the soil matrix porosity, *β* is the volumetric water compressibility, *p* is the incremental pore pressure with reference to the hydrostatic condition (overpressure), *x* and *z* are the horizontal and vertical coordinates. *Dp* refers to the total or Eulerian derivative, which can be treated as a partial time derivative ∂*p*/∂*t* by using a Lagrangian approach^24^ with a dynamic mesh where the grid nodes follow the grains in their consolidation movements. In this case, the second term of *Dp* = ∂*p*/∂*t* + *v*_*g*,*z*_ ⋅ ∂*p*/∂*z* vanishes and the mesh nodes move accordingly to the vertical grain velocity *v*_*g*,*z*_^51^:

4 $${v}_{g,z}(z,t)=\mathrm{(1}-\alpha {\sigma }_{z}){\int }_{0}^{z}\frac{(\alpha +{\sigma }_{z}\frac{\partial p}{\partial t})}{{\mathrm{(1}-\alpha {\sigma }_{z})}^{2}}dz$$

In Equation 4, *α* is the classical compressibility defined as *α* = *dε*_*z*_/*dσ*_*z*_ with *ε*_*z*_ the vertical deformaition, and linked to *c*_*b*_ by the relationship ${c}_{b}=\frac{pd\alpha \,/\,dp+\alpha }{1+\alpha p}$. It follows that the compaction *u*(*z*, *t*) (and hence the volumetric change) of the mesh elements can be computed as

5 $$u(z,t)=-\,{\int }_{0}^{z}\frac{\alpha {\sigma }_{z}}{1-\alpha {\sigma }_{z}}dz$$

Equation 3 holds under the following expression of the relative Darcy’s law

6 $$\varphi ({v}_{w,i}-{v}_{g,i})=-{k}_{i}\frac{{\rm{\partial }}\psi }{{\rm{\partial }}i},\,i=x,z$$

with *v*_*g*,*i*_ and *v*_*w*,*i*_ the (absolute) velocity of solid grains and water along the i direction, respectively, and *ψ* the hydraulic potential expressed as $\psi =z+{\int }_{0}^{p}dp/\gamma$. Notice that for the specific process of interest *v*_*g*,*x*_ is negligible and only vertical compaction is considered. Moreover, it is assumed incompressible solid grains and constant total stress expressed by Terzaghi’s principle in the form *σ*_*t*_ = *σ*_*z*,0_ + *σ*_*z*_ + *p*_0_ + *p*, with *σ*_*z*,0_ and *σ*_*z*_ the initial and incremental intergranular effective stress, respectively, and *p*_0_ the initial reference value for p.

To account for the delta progradation due to sediment accumulation, a sedimentation rate *ω*(*x*, *t*) is admitted and Equation 3 turns into^24^

7 $$\frac{\partial }{\partial x}(\frac{{k}_{x}}{\gamma }\frac{\partial p}{\partial x})+\frac{\partial }{\partial z}(\frac{{k}_{z}}{\gamma }\frac{\partial p}{\partial z})=(\frac{{\sigma }_{z}\frac{d\alpha }{d{\sigma }_{z}}+\alpha }{1-\alpha {\sigma }_{z}}+{\varphi }\beta )Dp-\frac{{\sigma }_{z}\frac{d\alpha }{d{\sigma }_{z}}+\alpha }{1-\alpha {\sigma }_{z}}D{\sigma }_{t}$$

In natural conditions, the variation of the total stress, *Dσ*_*t*_, is due to the change of load by new sediment deposition on the delta surface, thus *Dσ*_*t*_ = *ω*(*x*, *t*) (1 − *ϕ*_0_)(*γ*_*s*_ − *γ*) with *γ*_*s*_ the specific weight of the grains and *ϕ*_0_ the initial porosity at *σ*_*z*,0_. The material properties such as porosity, compressibility, and hydraulic conductivity are functions of the intergranular effective stress to account for their variability with the progressive deformation of the soil matrix. Indeed, *c*_*b*_, *k*_*z*_, and *ϕ* diminish at increasing values of *σ*_*z*_.

The numerical solution of Equations 5 and 7 is implemented in NATSUB-2D by a Finite Element discretization, using a back Euler method for the time integration and a fixed-point iteration scheme to solve the material and geometric non-linearities. A constant time step equal to 1 year has been adopted.

**Figure 7:**
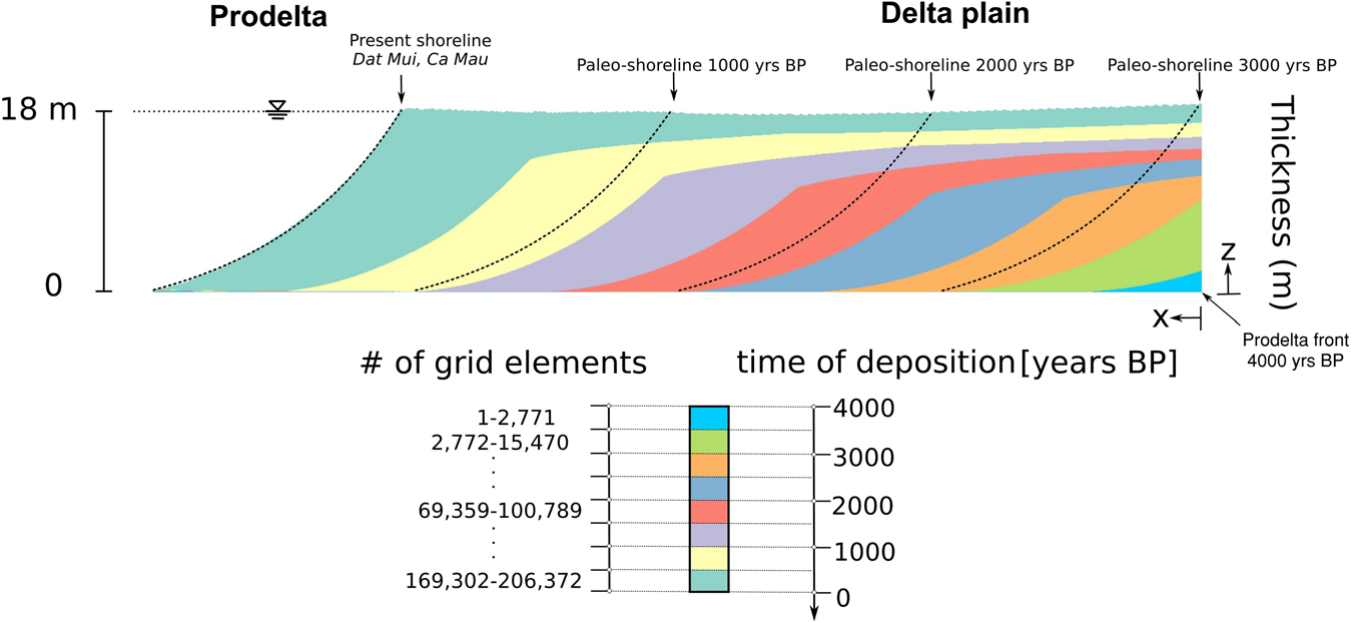
Evolution of the finite element mesh. The number of triangular elements in each temporal interval is provided. Note that the mesh size increases in time to account for deposition. The age of the grid elements is highlighted by colors.

Figure 7 shows the evolution of the computing mesh in time. The increasing number of triangular elements is provided to point out the spatio-temporal evolution of the computing grid following the delta formation and progradation.
